# IMPACT OF FAMILY FUNCTION ON THE RELATIONSHIP BETWEEN SELF-PERCEPTION OF AGING AND STRESS IN MIDLIFE

**DOI:** 10.1093/geroni/igz038.1718

**Published:** 2019-11-08

**Authors:** Hyun-E Yeom, Eunyoung Park, Misook Jung

**Affiliations:** Chungnam National University, Daejeon, Korea, Republic of

## Abstract

Midlife is a transitional period with considerable stress related to health changes and interpersonal responsibilities. Understanding how self-perception of aging and family function affect stress is essential to improve quality of life in midlife and beyond. The purpose of this study was to examine the association among self-perception of aging, family function, and stress with a specific focus on the interaction between self-perception of aging and family function, which affect stress in midlife Koreans. This is a cross-sectional study. Data on a convenience sample of 249 midlife Koreans (age mean= 50.6, 50.1% male) were collected through a self-administered survey and analyzed using the PROCESS macro for SPSS. Self-perception of aging was significantly related to family function (r= -.121, p=.045), and family function was related to stress (r= -.402, p<.000). Self-perception of aging was a significant predictor for stress (β= -.130, p=.008) after adjusting for age, gender, subjective health status, and chronic health problems. A significant interaction between family function and self-perception of aging on stress was found (β= -.261, p=.006), indicating that the influence of self-perception of aging on stress was different depending on family function. Self-perception of aging was a strong predictor of stress in individuals who reported poorer support from family members, but not in those who reported better support. Our findings emphasize the importance of supportive family function, which could regulate the impact of self-perception of aging on stress in midlife. Developing psycho-cognitive interventions to improve self-perception of aging and supportive interaction between family members is warranted.

